# Leptospirosis as an important differential of pulmonary haemorrhage on the intensive care unit: a case managed with VV-ECMO

**DOI:** 10.1186/s40560-020-00447-2

**Published:** 2020-04-26

**Authors:** James Barnacle, Stefan Gurney, Stephane Ledot, Suveer Singh

**Affiliations:** 1 Department of Infectious Diseases, St Mary’s Hospital, Imperial College Healthcare NHS Trust, London, UK; 2 Intensive Care Unit, Bristol Royal Infirmary, University Hospitals Bristol NHS Trust, Bristol, UK; 3 Adult Intensive Care Unit, Royal Brompton Hospital, Royal Brompton and Harefield NHS Trust, London, UK

**Keywords:** Leptospirosis, Extra-corporeal membrane oxygenation, Pulmonary haemorrhage, Diffuse alveolar haemorrhage, Weil’s disease

## Abstract

**Background:**

Leptospirosis is a potentially fatal zoonosis. It can cause a wide range of symptoms, including diffuse alveolar haemorrhage which occurs in a minority of cases but carries a mortality of over 70%. These patients may present with severe acute respiratory failure. The differential diagnosis for diffuse alveolar haemorrhage is broad whereas prompt diagnosis and treatment can be lifesaving.

**Case presentation:**

A 20-year-old previously fit and well trout farm worker presented with a 3-day history of malaise, fevers, diarrhoea, vomiting and jaundice. He developed haemoptysis, severe headaches, neck stiffness and photophobia on the day of emergency admission. He was anaemic and thrombocytopenic. Anuric acute kidney injury (urea 32, creat 507) required immediate haemofiltration. In view of progressive respiratory failure with four-quadrant lung infiltrates on imaging, he was given broad spectrum antibiotics and pulsed methylprednisolone empirically, in case of a vasculitic pulmonary-renal presentation. He was intubated within 48 h of admission. Despite attempted protective ventilatory management, he remained hypoxaemic and developed pneumomediastinum. He was retrieved to a specialist cardiorespiratory intensive care unit on femoro-femoral mobile VV-ECMO. Three days from admission, results showed positive *Leptospira* IgM and real-time PCR. Serial bronchoscopies showed old and fresh clots, but not the classical progressive late red tinge of the returned lavage fluid. After eight days, VV-ECMO was weaned, he was extubated three days later, and made a full recovery. At 9 months follow-up, he was clinically better, with resolution of the CT scan findings and near normal lung function, albeit with low normal gas transfer.

**Conclusions:**

Leptospirosis is a rare but important differential to be considered in diffuse alveolar haemorrhage presenting to the ICU, especially in young males. A thorough history for occupational or recreational risk factors may offer the diagnostic clue. Most patients recover fully with antibiotics. However, resulting acute severe respiratory failure can ensue. In this situation, early consideration for respiratory ECMO support offers time for clearance of endobronchial clot, parenchymal recovery, and prevention of ventilator-induced lung injury. Steroids have no clear evidence but may be used to avoid delay in treating suspected vasculitic or autoimmune causes of diffuse alveolar haemorrhage.

## Background

Leptospirosis is a zoonotic bacterial infection caused by the spirochaete *Leptospira* spp. Public Health England reported 92 lab-confirmed cases of leptospirosis in the UK in 2017 [1]. Transmission usually occurs through contact with the urine of infected rodents. Symptoms are frequently mild and flu-like, although severe complications may occur. Jaundice and renal failure secondary to leptospirosis is termed Weil’s disease after the 19^th^ Century German physician Adolf Weil. Renal failure tends to be non-oliguric and reversible.

Diffuse alveolar haemorrhage (DAH) occurs in approximately 3.7% of leptospirosis cases and is the major cause of death, with mortality rates exceeding 70% [2]. The differential diagnosis is broad and either pulmonary capillaritis, bland pulmonary haemorrhage, or diffuse alveolar damage may be seen on histology [3]. Traditionally, severe bleeding has been a relative contraindication to extra-corporeal membrane oxygenation (ECMO), which requires systemic anticoagulation to maintain circuit patency. However, cases have been reported of DAH successfully managed using ECMO [4].

Here, we present a case of leptospirosis presenting as DAH and multi-organ failure, requiring support with veno-venous ECMO (VV-ECMO). This case demonstrates the importance of considering rarer causes such as leptospirosis amongst the differentials of severe acute respiratory failure due to pulmonary haemorrhage.

## Case presentation

**Fig. 1:**
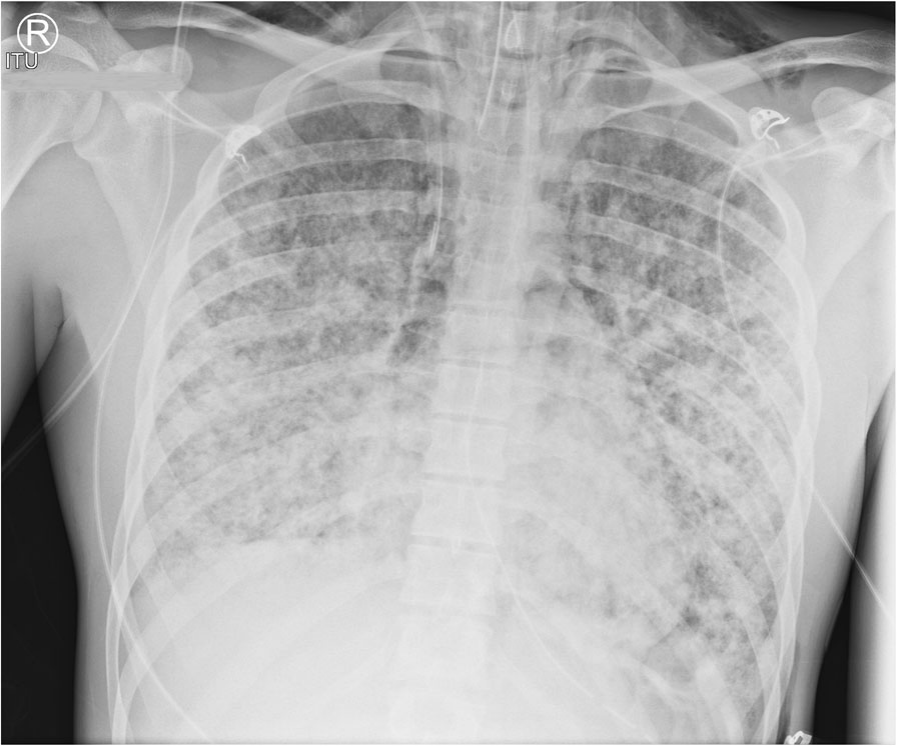
Chest X-ray of patient on admission to local hospital post-intubation

A 20-year-old male trout farm worker presented to his local hospital with a 3-day history of malaise, fevers, diarrhoea, vomiting and jaundice. He developed haemoptysis, severe headaches, neck stiffness and photophobia, leading to emergency admission. There were no rashes, swellings, nor melaena. He had no significant past medical history and was taking no prescription medications. He was an occasional smoker and alcohol drinker. He remained hypoxic despite supplemental oxygen and after a short admission to the high-dependency unit he was intubated and ventilated in the intensive care unit (ICU). A post-intubation chest x-ray showed four-quadrant airspace ‘ground glass’ opacification (Fig. 1). His admission bloods demonstrated haemoglobin (Hb) 99 g/l, platelets 25,000/μl, white blood cells (WBC) 10,400/μl, C-reactive protein (CRP) 195 mg/dl, creatinine 392 μmol/l, urea 17 mmol/l and total bilirubin 117 μmol/l. HIV, autoimmune, vasculitis and atypical pneumonia blood and/or urine screens were negative.

Empirical ceftriaxone was initiated following sequential escalating regimens of amoxicillin, gentamicin, metronidazole, then piperacillin-tazobactam and levofloxacin, following local microbiology advice. Intravenous doses of 1 g methylprednisolone daily for two days were initiated to treat the possibility of vasculitis associated pulmonary haemorrhage, pending results. He received early renal replacement therapy for acute kidney injury (AKI). A high-resolution CT scan of the chest after intubation demonstrated pneumomediastinum, pneumoperitoneum and extensive dependent consolidation. Due to the risk of worsening pneumothoraces and ventilator-induced lung injury (VILI) on optimised conventional ventilatory management, and concerns about the risk of prone positioning in this context, he was accepted for retrieval on the national severe acute respiratory failure (SARF) pathway. At the time of referral, he had SpO_2_ 94%, pO_2_ 11.4 kPa, pCO_2_ 6.5 kPa and pH 7.29 on pressure-controlled ventilation with FiO_2_ 1.0, positive end-expiratory pressure (PEEP) 15 cmH_2_O, total inspiratory pressure (*P*_insp._) 30 cmH_2_O, tidal volume (*V*_T_) 450 ml, compliance 30 ml/cmH_2_O, PaO_2_/FiO_2_ ratio 86mmHg and a Murray score of 3.75. He was commenced on VV-ECMO (using a standard bi-femoral configuration: 25-French access cannula and a 23-French return cannula) at 2930 revolutions per minute (RPM), generating a blood flow rate of 4.7 L/min with a sweep gas flow of 5.0 L/min of 100% oxygen to facilitate lung protective ventilation.

**Fig. 2:**
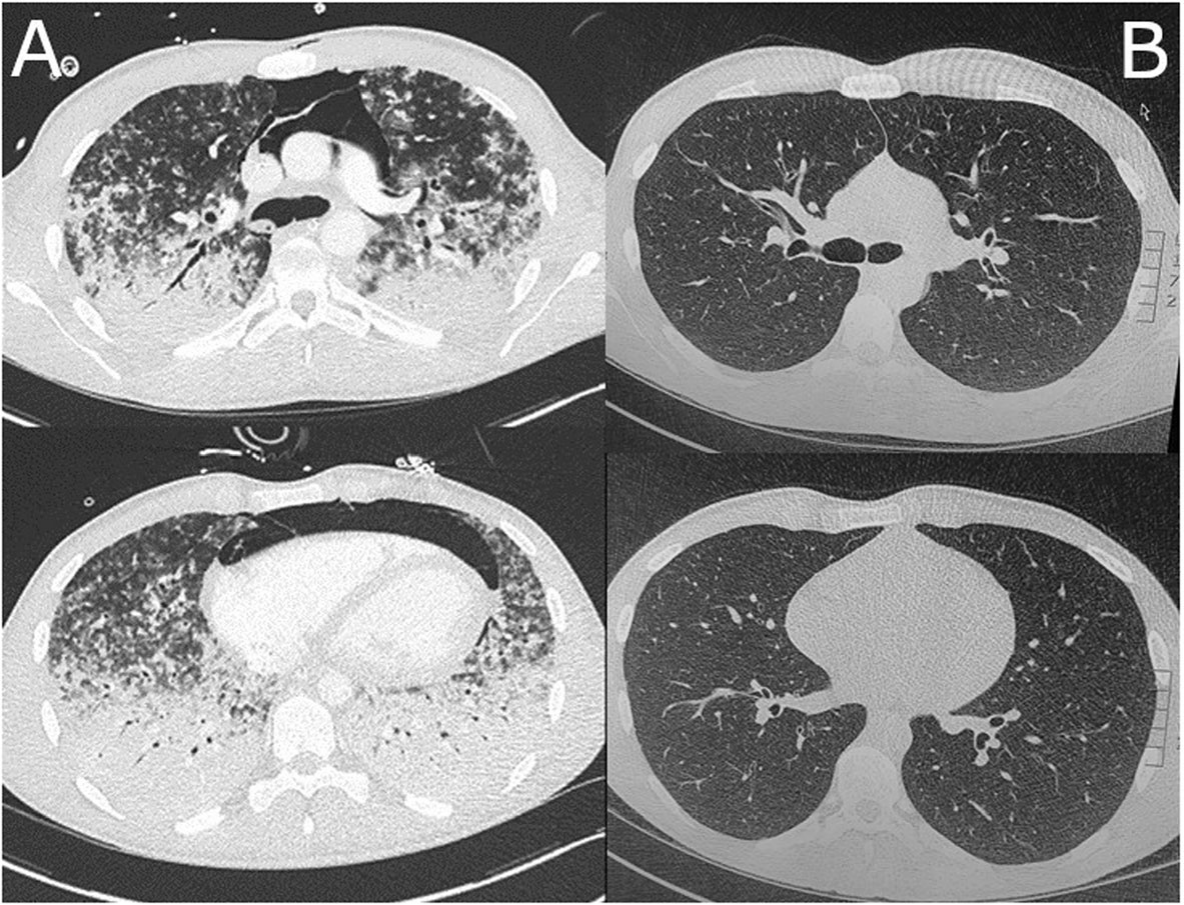
High-resolution CT images on day 1 of ECMO (**a**) and 9 months post-discharge (**b**)

At this ECMO centre, a further high-resolution CT scan was performed demonstrating persistent consolidation with pronounced branching nodularity and extensive mediastinal emphysema (Fig. 2a) with a normal CT head, abdomen and pelvis. The pneumomediastinum prevented a full echocardiographic examination but subcostal windows demonstrated preserved left ventricular function, non-dilated ventricles and no significant mitral or tricuspid regurgitation. On day 1 of his time on ECMO, positive serum *Leptospira* Panbio IgM ELISA and dual-target real-time 16SrRNA/Lipl32 polymerase chain reaction (PCR) results became available, confirmed by the reference laboratory, to which Public Health England was alerted. Further tests including blood β-d-glucan and galactomannan, as well as cultures, mycoplasma, viral PCR screen and acid-fast bacilli (AFB) from broncho-alveolar lavage (BAL) were all negative.

*Top images at the level of the carina; bottom images at the level of the superior pulmonary veins

The patient was kept on lung-protective ventilation using bi-level positive airway pressure (BiPAP) with PEEP 5 cmH_2_O, *P*_insp._ 12 cmH_2_O and *V*_T_ 250–300 ml, rate 10/min. He was sedated and paralysed with a cis-atracurium infusion for the first 24 h. He received low-dose noradrenaline to keep his mean arterial pressure (MAP) above 65 mmHg but no inotropic support. Haemodiafiltration with regional citrate-based anticoagulation was required until day seven of the admission. Methylprednisolone was continued at a maintenance dose of 60 mg bd for 4 days until the full vasculitic screen had come back negative, with fluconazole added prophylactically in view of the steroid burden. Ceftazidime and gentamicin were started empirically for rising inflammatory markers and fever on day 6 of ECMO and continued for 7 days. The patient received 8 days of ECMO support. His coagulation studies were normal throughout the admission, but his platelets were 56,000/μl on arrival, with a prior nadir of 25,000/μl. Systemic heparinisation was therefore initiated cautiously with a target anti-Xa level of 0.2–0.3 IU/ml according to local policy.

Serial bronchoscopic examination by an experienced bronchoscopist initially showed old clots in the lower lobes bilaterally but on day 3 of the ECMO run, fresh blood was seen with mucosal bleeding points in the apical segments of the right and left lower lobes; these were treated with 2 ml of 1:100,000 adrenaline to achieve haemostasis. There was no central airway source of bleeding on careful inspection. His anti-Xa target was reduced to 0.2 IU/ml and tranexamic acid 1 g IV bd was commenced. A liver ultrasound was normal, hepatitis A and E serology was negative and a review from hepatology confirmed that the enzyme picture fitted leptospirosis and treatment should be supportive.

On day 9 at the Royal Brompton Hospital, the patient was decannulated from ECMO whilst still receiving lung protective ventilation. The patient was extubated three days post-ECMO decannulation. A post-decannulation vascular ultrasound revealed no deep vein thromboses and he was treated with 5000 units of unfractionated heparin subcutaneously twice daily, in view of his abnormal renal function and restored platelet counts. He was extubated 3 days after decannulation from ECMO and repatriated to his local hospital where he made a full recovery.

At 9 months follow-up, he was clinically better, with resolution of the CT scan findings (Fig. 2b), a normal 6-min walk test distance > 400 m, and near normal lung function, albeit with low normal gas transfer (FEV1 5.68 L, > 100%, VC 6.7 L, > 100%, TLCO 79.8%, KCO 74.2%).

## Discussion

### Diffuse alveolar haemorrhage

Diffuse alveolar haemorrhage is caused by disruption of the alveolar-capillary basement membrane. It can have a mortality exceeding 50% in the intensive care setting and is traditionally associated with haemoptysis, although this may be absent in a significant proportion of patients [5]. Radiological features include a non-specific diffuse infiltrative opacification pattern on chest X-ray and ground-glass opacities on CT in acute bleeding [6]. It carries a wide differential diagnosis including vasculitis, infection, drugs, acute respiratory distress syndrome and heart failure [3]. In leptospirosis, pulmonary haemorrhage has been the presenting sign preceding other clinical features [7]. In patients admitted to the ICU with undifferentiated pulmonary haemorrhage, leptospirosis should be considered even in the absence of other typical features. DAH does not always present with classical BAL findings, as was the case here. There was evidence of clotted appearances interspersed with fresh blood. This was probably related to the delay between initial presentation with haemoptysis and the BAL, which was only performed after the initiation of ECMO. By this time, some interalveolar and endobronchial clotting would have commenced. It remains speculative as to whether the classical BAL findings would have been found if BAL was performed at the referring centre at presentation or soon after intubation. However, we did not find an alternative source, and no alternative microbiology became apparent from the ECMO BAL samples.

### Leptospirosis

Leptospirosis is a zoonotic disease caused by the aerobic spirochaete *Leptospira* spp. It is spread through contact with the urine of mammals, usually rodents. Portals of entry include skin abrasions, mucous membranes or conjunctivae; it is unclear whether the bacteria can penetrate intact skin. Diagnosis is usually made using serology tests including IgM enzyme-linked immunosorbent assay (ELISA). However, rapid diagnostics by blood PCR are also available [8]. *Leptospira* can be cultured but it requires special media. Growth is observed beyond a week and may take up to 3 months meaning routine blood cultures will be negative [9].

Clinically, leptospirosis is a biphasic illness with an acute febrile leptospiraemic phase lasting about a week, followed by an immune leptospiruric phase characterised by antibody production during which most of the complications occur. The average incubation period for leptospirosis is 10 days. Most cases involve a mild, flu-like, self-limiting illness or may be subclinical. However, there are many clinical manifestations. Fever, myalgia and headache are almost universal. In addition, conjunctival suffusion, non-productive cough, abdominal pain, nausea, vomiting and diarrhoea are commonly seen in over one third of patients [9]. The mortality for anicteric leptospirosis is very low. Neurological complications may be seen, the most common of which is aseptic meningitis with a lymphocytic predominance. Laboratory studies are non-specific. White cells are typically normal, and thrombocytopenia may occur.

Icteric leptospirosis, or leptospirosis with jaundice, occurs in 5–10% of cases and heralds a more severe clinical course. Weil’s disease is the term used for leptospirosis complicated by jaundice and renal failure. The jaundice is not associated with hepatic necrosis and any liver dysfunction returns to normal after recovery. Renal failure tends to be non-oliguric and recovery is generally complete, although renal replacement may be required in the acute phase. Mortality for Weil’s disease in the UK is around 5% [10].

Treatment of leptospirosis is mainly supportive. The efficacy of antibiotics remains unclear. A Cochrane review demonstrated a non-significant trend towards shortening the duration of clinical illness only [11]. In practice, antibiotics are recommended, especially for severe disease. Leptospirosis is susceptible to a wide range of antibiotics and resistance does not seem to be a problem. Traditionally treatment is with a penicillin or doxycycline, but cephalosporins or azithromycin are acceptable alternatives. When comparing penicillin, doxycycline and cephalosporins, no benefit has been found in using one over another [11].

### ECMO support in leptospirosis

There are 10 other reports of the use of ECMO in the management of leptospirosis: 9 veno-venous, and 1 veno-arterial (Table 1). Common characteristics include male gender and young age (average 39 years, 20 to 72 years). The duration of ECMO was 2–18 days in survivors, typically 1 week. All patients received at least a penicillin or cephalosporin. Almost all patients had a convincing transmission source, either occupational or recreational, which highlights the importance of a thorough collateral history in suspected cases of leptospirosis. Including this case, mortality was 1/11 (9.1%) amongst ECMO patients at discharge, which is much lower than the reported mortality of over 50% in acute respiratory failure in an ICU context without ECMO [2, 12].

**Table 1:** Cases in the literature reporting the use of VV-ECMO in leptospirosis

	Country	Age (sex)	Transmission	Treatment	Time from admission to ECMO	ECMO modality	ECMO duration (days)	Outcome
Our patient	UK	20 (M)	Trout farmer	Levofloxacin Ceftriaxone Doxycycline Methylpred.	4 days	VV	8	Full recovery
Chaikajornwat et al. (2020) [13]	Thailand	39 (M)	Street vendor (presumed floodwater exposure)	Levofloxacin Ceftriaxone Methylpred.	12 h	VV	8	Full recovery
Cantwell et al. (2017 )[14]	Chile	39 (M)	Working in a contaminated drain with mice	Ceftriaxone Metronidazole Meropenem Vancomycin Amikacin	1 day	VV	8	Full recovery
Kutlesa and Gjurasin (2017) [15]	Croatia	72 (F)	Daily contact with bulls	Ceftriaxone Flucloxacillin Methylpred.	1 day	VV	6	Full recovery
Ludwig et al. (2017) [16]	Germany	34 (M)	Pet rat	Tazocin Ciprofloxacin Prednisolone	12 h	VV	<1	Died
Pardinas et al. (2017) [17]	USA	32 (M)	Freshwater swimming	Ceftriaxone Doxycycline	2 days	VV	18	Full recovery
Umei and Ichiba (2017) [18]	Japan	50 (M)	Occupational (sushi chef)	Ceftriaxone Levofloxacin Minocycline Vancomycin Meropenem Benzylpenicillin RHZE	3 days	VV	11	Full recovery
Héry et al. (2015) [19]	Laos	38 (M)	Visiting rice fields	Ceftriaxone Doxycycline Amoxicillin	< 1 day	VV	9	Full recovery
Liao et al. (2015) [20]	Taiwan	32 (M)	Walking barefoot through water at fruit market	Penicillin G Levofloxacin	1 day	VV	6	Full recovery
Kahn et al. (2006) [21]	Austria	?? (M)	Unknown	Augmentin Clarithromycin Imipenem Ciprofloxacin	Unknown	VA	2	Full recovery
Arokianathan et al. (2005) [22]	UK	30 (M)	Clearing sewage (bare hands with abrasions)	Cefuroxime Clarithromycin Metronidazole Benzylpenicillin	Unknown	VV	7	Full recovery

*VV* veno-venous, *VA* veno-arterial, *RHZE* rifampicin, isoniazid, pyrazinamide, ethambutol

Leptospirosis with DAH appears to carry a high risk of barotrauma [19, 23, 24] The current case was complicated by pneumomediastinum. One report of acute respiratory failure managed without ECMO reported spontaneous pneumomediastinum and recurrent airway occlusion due to haemorrhage and required a six-week ICU admission and tracheostomy [24]. Our patient had a much better clinical course despite the use of ECMO.

### Steroid treatment in leptospirosis with diffuse alveolar haemorrhage

The evidence for steroids in severe leptospirosis is insufficient to recommend their routine use. A meta-analysis found just four trials looking at steroid use [25], all of which had study limitations [26–29]. Our patient received two doses of 1 g methylprednisolone followed by 1 mg/kg maintenance to treat any possible underlying vasculitic process prior to the diagnosis. Ludwig et al. gave 1 g of IV prednisolone in combination with an extracorporeal cytokine absorbent filter shortly before their patient died [16]. Kutlesa and Gjurasin gave methylprednisolone for 10 days but the dose is not recorded [15]. Chaikajornwat et al. gave 250 mg methylprednisolone every 6 h for the first 2 days of their patient’s admission in case of vasculitis [13]. Further trials examining the role of steroids in leptospirosis are required, but in the context of pulmonary haemorrhage without a solid diagnosis, steroids should be considered to treat possible vasculitic causes.

## Conclusions

Leptospirosis is a rare but important differential to be considered in diffuse alveolar haemorrhage presenting to the ICU in acute respiratory failure, especially in young males. A thorough history for occupational or recreational risk factors is crucial and if in doubt leptospirosis serology and PCR should be sent. Most patients make a full recovery with treatment, but barotrauma does seem to be associated with DAH in leptospirosis and ECMO support should be considered early to avoid harm from non-protective ventilation. There is no clear evidence for steroids in confirmed leptospirosis and their use cannot be recommended. However, they are often instituted empirically prior to diagnosis in case of vasculitic causes for pulmonary haemorrhage. Unfortunately, leptospirosis continues to cause the greatest mortality and morbidity in low- and middle-income countries where access to ECMO is severely limited. In the future, the encouraging outcomes of patients managed with ECMO should be considered when arguing for the introduction of ECMO programmes in high-burden countries.

## Data Availability

Not applicable.

## Abbreviations

ADAMSTS13: A disintegrin and metalloproteinase with thrombospondin type 1 motif, member 13
AFB: Acid-fast bacilli
AKI: Acute kidney injury
BAL: Broncho-alveolar lavage
BiPAP: Bi-level positive airway pressure
CT: Computer tomography
DAH: Diffuse alveolar haemorrhage
ECMO: Extra-corporeal membrane oxygenation
ELISA: Enzyme-linked immunosorbent assay
FEV1: Forced expiratory volume over 1 second
Hb: Haemoglobin
ICU: Intensive care unit
IV: Intravenous
KCO: Transfer coefficient
MAP: Mean arterial pressure
pCO_2_: Carbon dioxide partial pressure
PCR: Polymerase chain reaction
PEEP: Positive end-expiratory pressure
PHE: Public Health England
*P*_insp_: Total inspiratory pressure
pO_2_: Oxygen partial pressure
RHZE: Rifampicin, isoniazid, pyrazinamide, ethambutol
RPM: Revolutions per minute
TLCO: Diffusing capacity for carbon monoxide
VC: Vital capacity
*V*_T_: Tidal volume
VV-ECMO: Veno-venous extra-corporeal membrane oxygenation
WBC: White blood cells
